# Dynamics of Tribofilm Formation in Boundary Lubrication Investigated Using In Situ Measurements of the Friction Force and Contact Voltage

**DOI:** 10.3390/ma17061335

**Published:** 2024-03-14

**Authors:** Anna E. Tsai, Kyriakos Komvopoulos

**Affiliations:** Department of Mechanical Engineering, University of California, Berkeley, CA 94720, USA

**Keywords:** additives, blend formulations, boundary lubrication, dispersants, friction, contact voltage, tribofilms, zinc dialkyl dithiophosphate

## Abstract

The complex dynamics of tribofilm formation on boundary-lubricated steel surfaces were investigated in real time by combining in situ measurements of the temporal variation of the coefficient of friction and contact voltage. Sliding experiments were performed with various blends consisting of base oil, zinc dialkyl dithiophosphate (ZDDP) additive, and two different dispersants at an elevated oil temperature for a wide range of normal load and fixed sliding speed. The evolution of the transient and steady-state coefficient of friction, contact voltage, and critical sliding distance (time) for stable tribofilm formation were used to evaluate the tribological performance of the tribofilms. The blend composition affected the load dependence of the critical sliding distance for stable tribofilm formation. Tribofilm friction was influenced by competing effects between the additive and the dispersants. Among various formulations examined, the tribofilm with the best friction characteristics was found to be the blend consisting of base oil, a small amount of ZDDP, and a bis-succinimide dispersant treated with ethylene carbonate. The results of this study demonstrate the effectiveness of the present experimental approach to track the formation and removal of protective tribofilms under boundary lubrication conditions in real time.

## 1. Introduction

The use of lubricating substances (solid or fluid) to control friction and wear is widespread in a variety of traditional and contemporary industries, including wind turbines, aerospace, automotive, power generation and transmission systems, manufacturing, electronics, photonics, microelectromechanical systems, and magnetic recording. Complex chemomechanical interactions between the polar molecules of base oils [1,2], fully formulated oils [3], and/or reactive additives [4] and the sliding surfaces lead to the formation of thin surface layers referred to as tribofilms. The resulting reactions are influenced by the chemical composition of the fluid lubricant and the sliding surfaces as well as various operation parameters, such as contact load, coefficient of friction (shear surface traction), relative sliding speed, sliding distance (time), interfacial temperature, and environmental conditions [5]. The main functionality of tribofilms is to control the friction and wear properties of interacting surfaces, often protecting them in a sacrificial manner. Because tribofilms play a critical role in the performance and longevity of a wide range of tribosystems, their formation, durability, and tribological properties have been the objective of numerous studies.

The chemical additives incorporated in base oil encompass a major class of lubricants leading to the formation of low-friction, antiwear tribofilms. Nonpolar base oils mainly function as lubricants, removing heat and wear particles and maintaining low friction [6], also acting as carriers of the additives and dispersants—surfactants used to prevent the aggregation of suspended particles. Current oil formulations include different types of additives demonstrating various functionalities. Zinc dialkyl dithiphosphate (ZDDP) is the antiwear/antioxidant additive most regularly incorporated in engine oils [7]. Since the introduction of ZDDP several decades ago, its kinetics of formation and removal [4,8,9,10,11], composition [12], and tribological properties [13,14] have been extensively investigated. ZDDP is predominantly decomposed by hydrolysis or thermolysis, ultimately forming a tribofilm containing zinc polyphosphate and a mixture of alkyl sulfides [8], which reduces friction and prevents wear of the metallic surfaces sliding in the boundary lubrication regime through rapid self-replenishment [9]. It has been reasoned that ZDDP formation is controlled by a stress-induced thermal activation process wherein localized shear stresses arising at asperity contact interfaces lower the thermal activation energy for ZDDP formation and increase the reaction rate [11]. Under high temperature and contact pressure, the ZDDP molecules decompose following first-order reaction kinetics, with the rate of tribofilm formation demonstrating an exponential dependence on both temperature and contact pressure, consistent with stress-assisted reaction rate theory [15,16]. In fact, an investigation of the effects of mechanical stresses on chemomechanical reactions of ZDDP-containing oils revealed that tribofilm growth was predominantly driven by shear (friction) stresses, whereas compressive stresses hindered growth [17]. Thus, tracking the evolution of the friction force and the contact voltage in real time may provide useful insight into the dynamics of tribofilm formation and removal. However, despite the excellent antiwear and antioxidant properties of ZDDP, this additive also has some drawbacks. Most importantly, it can lead to ash formation in the exhaust during combustion, reducing the efficiency of emissions-control catalytic converters, it may result in sludge formation that could impede lubricant circulation, and its long half-life raises environmental concerns. For this reason, the demand for reduced amounts of zinc-based additives or alternative additives that can offer the same protection to metal surfaces without the foregoing drawbacks has significantly increased in recent years. As a result, molybdenum dialkyl dithiocarbamate (MoDTC) was introduced as another promising additive that can form a low-friction tribofilm [4,13] possessing a lattice structure consisting of MoS_2_ nanosheets, although it has been reported that MoS_2_ tribofilms are much easier to remove than ZDDP tribofilms [18]. Effective low-friction/antiwear tribofilms have also been produced from phosphorous- and sulfur-containing ionic liquids [4,19,20,21] and ceramic nanocrystals [22], through the synergy of transition metal oxide nanoparticles and conventional sulfur-containing oil additives [23], borate-modified blends [24], and borate-, phosphorous-, and sulfur-containing white oil and gear oil [25,26,27]. The good stiffness and strength, easy-to-shear ability, and potential to form antiwear tribofilms of two-dimensional transition metal carbides, nitrides, and carbonitrides, collectively known as MXenes, have also made them attractive additive candidates for tribological applications [28,29,30].

A balance of the additives used in the formulation of synthetic lubricants is crucial to successfully controlling friction and wear [6]. A concerted interaction that enhances the lubricant performance may occur when active additives are blended together. Alternatively, additives may compete for adsorption sites on the sliding surfaces, producing an unfavorable effect on the tribological properties. Previous studies have shown the importance of the antagonistic roles of ZDDP and dispersants. For example, succinimide dispersants have been reported to strongly interact with ZDDP [31]. Therefore, understanding the cooperative and contrasting effects between additives and dispersants is of particular importance to the development of new synthetic lubricants.

Even though the foregoing studies provided insight into the end-of-test tribofilm formation, not much is known about the dynamics of tribofilm formation, particularly the complexities associated with variations in applied load and oil formulation. Consequently, the principal objective of this study was to investigate how the temporal variation in tribofilm formation under boundary lubrication conditions can be tracked in real time. Thus, sliding experiments were performed at an elevated temperature with steel samples lubricated with various oil formulations containing base oil, reduced-concentration additive, and dispersant. The efficacy of different blends to reduce friction are interpreted in the context of experimental results showing the temporal variation in the coefficient of friction and contact voltage and measurements of the critical distance (time) for stable tribofilm formation at steady-state sliding in the boundary lubrication regime.

## 2. Experimental Methods

### 2.1. Lubricant Formulations

To examine the efficacy of the implemented experimental approach to discern the dynamics of tribofilm formation from different lubricant formulations, experiments were performed with blends consisting of base oil, various amounts of ZDDP, and a specific dispersant (Chevron Oronite Co., Richmond, CA, USA). Specifically, blend 1 was an additive-free lubricant consisting of base oil (100N), which was used as the reference blend. Blends 2 and 3 consisted of base oil and ZDDP in reduced concentrations, i.e., 0.05 and 0.08 wt%, respectively, corresponding to industrial standards of the phosphorus content in engine oils. Blends 4 and 5 consisted of base oil containing 0.1 wt% nitrogen-dispersant A and 0.1 wt% nitrogen-dispersant B, respectively. Dispersant A was a bis-succinimide treated with ethylene carbonate [32], whereas dispersant B had improved dispersancy and was produced by reacting a copolymer with at least one ether compound and at least one aromatic amine [33]. The preferred compound structures for dispersant B are given elsewhere [33]. Aside from structural differences, dispersant A had a lower molecular weight than dispersant B. Blends 6 and 7 consisted of base oil and mixtures of 0.05 wt% ZDDP and 0.1 wt% nitrogen-dispersant A or 0.1 wt% nitrogen-dispersant B. The chemical compositions of blends 1–7 are given in Table 1. The amount of nitrogen in blends 4 and 6 was significantly higher than that in blends 5 and 7. A comparison of the elemental contents given in Table 1 shows a significant concentration of P, Zn, and S in blends 6 and 7, attributed to the presence of ZDDP.

### 2.2. Specimens

Commercially available ball bearings and flat disks consisting of AISI 52100 steel (Falex Co., Sugar Grove, IL, USA) were used as specimens. The chemical composition of the steel specimens comprised 1.04% C, 0.35% Mn, 0.275% Si, 1.45% Cr, and 96.89% Fe (all wt%). The mechanical properties of the ball and disk specimens (provided by the manufacturer) are given in Table 2. Each disk specimen was polished with a metallographic polishing wheel (Buehler, Lake Bluff, IL, USA). First, the disks were polished with 400- and 600-grit SiC abrasive paper and then with 0.3 μm Al_2_O_3_ particles supplied on a polishing cloth. The average roughness *R_a_* of the mirror-polished disks was measured using a mechanical stylus profilometer (Dektak IID, Veeco Instruments, NY, USA) with a vertical resolution of 0.1 nm. A total of 12 polished disks were used to measure the *R_a_* roughness at 5 random locations on each disk surface. The *R_a_* roughness of the polished disks, obtained as the average of 60 roughness measurements, was found to be equal to 21.1 ± 7.9 nm. The ball bearings were not polished. Before testing, both the polished disks and the ball bearings were cleaned with hexane and acetone to remove any surface contaminants. To prevent surface changes due to environmental effects, the cleaned specimens were fully immersed in pure base oil until testing.

### 2.3. Friction Experiments

**Figure 1:**
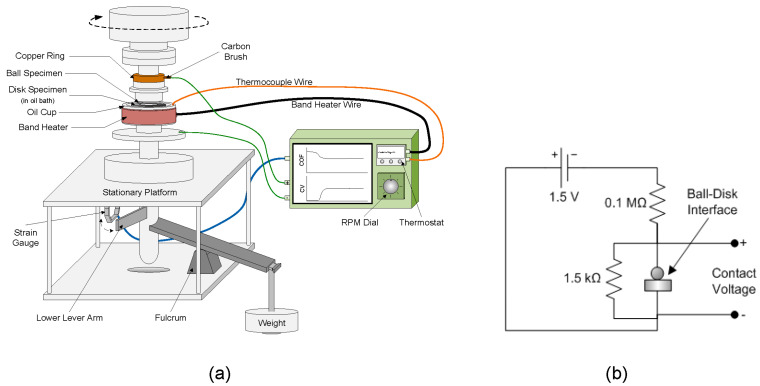
Schematics of (**a**) ball-on-disk tribometer and (**b**) electrical circuit used for in situ contact voltage measurement.

The tribological properties in the presence of each blend were evaluated with a modified ball-on-disk tribometer (Falex Co., Sugar Grove, IL, USA), shown schematically in Figure 1a, following the procedure of the standard ASTM G99-17 (ASTM International, West Conshohocken, PA, USA). The 0.8-cm-diameter ball specimen was press-fit into a holder attached to an electrically isolated rotating shaft. The 3.2-cm-diameter disk specimen was then placed on a pedestal enclosed by a circular cup that contained the lubricant. Rotation of the disk during testing was prevented by a pin on the pedestal that mated with the hole at the bottom of the disk specimen. O-rings were used at various joints of the oil cup to prevent oil leakage. During testing, the contact interface of the two specimens was fully immersed into the lubricant. A band heater (Tempco Electric Heater Co., Wood Dale, IL, USA) wrapped around the oil cup was used to heat the oil bath for ~18 min to a uniform temperature of 100 °C, which was maintained constant within ±5 °C during testing. Each blend was tested under a normal load *L* equal to 1.22, 2.45, 5.02, 7.49, and 10.15 kg. All tests were performed at a constant rotational speed of ~178 rpm, corresponding to a linear sliding velocity of 0.19 m/s. Calculations showed that the combination of these load and speed conditions resulted in sliding in the boundary lubrication regime. The duration of each test was set at 2 h. For statistical analysis, a minimum of 5 tests per blend were performed for a given load.

### 2.4. Coefficient of Friction and Contact Voltage Measurements

The friction force (determined as the ratio of the frictional torque measured by the strain gauges to the radial distance from the center of the wear track to the spindle axis) and the contact voltage were monitored during testing at a sampling frequency of 0.5 Hz. The frictional torque was measured with a Wheatstone strain-gauge bridge. To ensure the accuracy of the voltage output, the strain-gauge bridge was frequently calibrated with known torques. The coefficient of friction was calculated as the ratio of the determined friction force to the normal load applied as a dead weight.

The contact voltage across the ball–disk interface was measured with a voltage divider circuit, shown schematically in Figure 1b. Monitoring the contact voltage in situ has been proven to be an effective method for studying the formation of nonconductive tribofilms at sliding contact interfaces [34]. Because of the significantly lower restriction resistance produced by the surface roughness compared to that due to electron tunneling through an insulating tribofilm formed at the contact interface, very low contact voltages (~0 V) indicated direct metal-to-metal contact, whereas contact voltages of the order of several mV suggested the development of an insulating tribofilm. A steady-state contact voltage of ~22 mV indicated the formation of a stable nonconductive tribofilm.

## 3. Results and Discussion

In this section, results from in situ coefficient of friction and contact voltage measurements are presented and discussed for a wide load range and different oil formulations to demonstrate the effectiveness of the experimental approach to capture the dynamics of tribofilm formation in real time.

### 3.1. Coefficient of Friction and Contact Voltage

**Figure 2:**
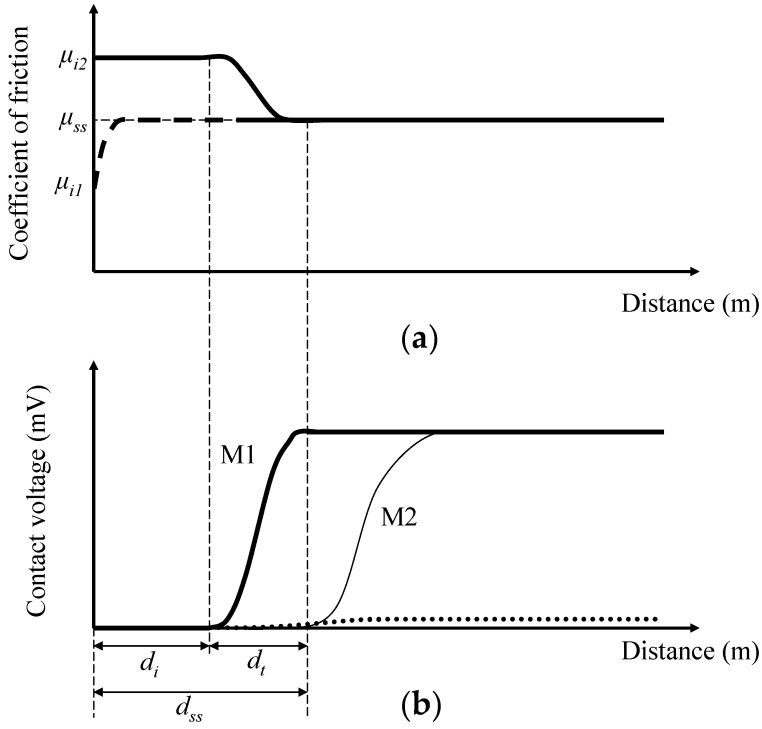
Schematics of (**a**) coefficient of friction and (**b**) contact voltage responses.

The variations in the coefficient of friction and contact voltage with the sliding distance and load due to each blend are discussed first. Each data point represents the average of five measurements obtained from 50 m intervals of sliding distance under the same test conditions. Therefore, each data point includes the experimental scatter within a given test and from test to test. Coefficient of friction and contact voltage plots with standard deviation error bars performed under the same conditions can be found in Section I of Supplementary Materials. Figure 2 shows schematics of typical coefficient of friction and contact voltage temporal responses. In most experiments, the initial coefficient of friction was between *μ_i_*_1_ ≈ 0.10 and *μ_i_*_2_ ≈ 0.14, reaching a steady state *μ_ss_* after a certain sliding distance, depending on blend and load (Figure 2a). For *L* < *L_c_*, where *L_c_* is the critical load, the contact voltage generally exhibited a three-stage response comprising incubation, transient, and steady-state stages with corresponding sliding distances denoted by *d_i_*, *d_t_*, and *d_ss_* (Figure 2b). In the incubation stage, the contact voltage fluctuated about the zero level, indicating the absence of an insulating tribofilm at the contact interface. The instigation of the transient stage was characterized by a notable rise of the contact voltage, often accompanied by abrupt fluctuations due to the competing processes of tribofilm formation and removal. A steady-state contact voltage was reached when tribofilm formation dominated the removal process. For some test conditions, a nonzero steady-state coefficient of friction was obtained for the same distance *d_ss_*, delineating the end of the transient contact voltage response (Figure 2b), hereafter referred to as mode M1. For *L* > *L_c_*, the contact voltage did not reach a distinguishable steady state; instead, it continued to fluctuate slightly above the zero level (Figure 2b), hereafter referred to as mode M2. For *L* ≈ *L_c_*, some test conditions yielded a bimodal behavior of the contact voltage. For about half of these tests, the contact voltage response indicated tribofilm formation (mode M1), whereas for the other half of the tests, the contact voltage revealed that stable tribofilm formation did not occur under the particular test conditions (mode M2). Results of the various sliding stages for each blend and different loads are given in Section II of Supplementary Materials.

**Figure 3:**
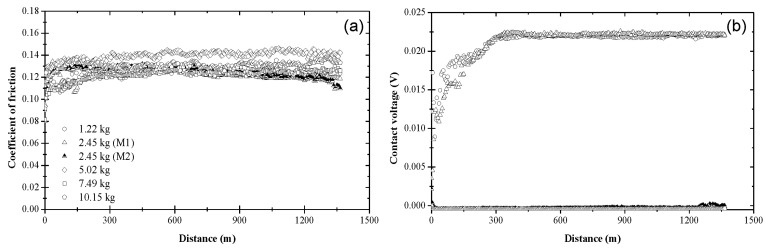
(**a**) Coefficient of friction and (**b**) contact voltage versus sliding distance and load for blend 1 (base oil).

Figure 3 shows the variation of the coefficient of friction and contact voltage with the sliding distance and the load due to blend 1. As shown in Figure 3a, the coefficient of friction increased from an initial value in the range of 0.10–0.13 to a slightly higher steady-state value between 0.11 and 0.14. Although a slightly higher coefficient of friction was obtained for a 5.02 kg load, the data do not display a consistent load effect on the friction behavior of the steel surfaces lubricated with base oil. Figure 3b indicates the formation of an electrically insulating film (apparently an oxide film since the surfaces were lubricated with base oil) at the sliding interface for *L* = 1.22 kg. A steady-state contact voltage of ~0.022 V was measured after sliding for a distance of ~300 m under this load. The increase in contact voltage during the transient stage (*d_t_* ≈ 300 m) under the lightest load may be associated with complex surface activation and deformation processes, which affected the mechanisms of tribofilm formation and removal. The nonzero steady-state contact voltage can be attributed to a critical tribofilm thickness for full coverage of the real contact area. A bimodal contact voltage response was obtained for *L* = 2.45 kg, suggesting that the critical load for base oil is close to 2.45 kg. For this load, about half of the tests yielded a contact voltage response similar to that obtained with the lightest load (mode M1), whereas the other half of the tests produced a zero contact voltage throughout testing (mode M2). For higher loads (*L* > 2.45 kg), the contact voltage exhibited fluctuations about the zero level during the entire test duration, indicating the dominance of tribofilm removal (mechanical wear) over tribofilm formation (tribochemical reactions).

**Figure 4:**
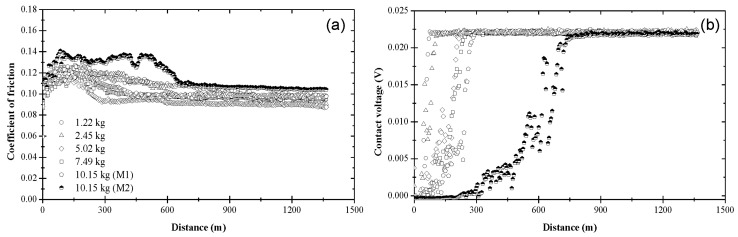
(**a**) Coefficient of friction and (**b**) contact voltage versus sliding distance and load for blend 2 (base oil + 0.05 wt% ZDDP).

The variation of the coefficient of friction and contact voltage with sliding distance and load due to blend 2 is displayed in Figure 4. The coefficient of friction increased from an initial value in the range of 0.09–0.12 to a slightly higher value after the first 150–200 m of sliding, eventually reaching a steady state between 0.09 and 0.10 (Figure 4a), which is lower than that obtained with base oil (Figure 3a). While the load did not affect the magnitude of the steady-state coefficient of friction significantly, it affected the sliding time (distance) for reaching steady state. As seen in Figure 4a, the general trend was for the sliding distance (time) for steady-state tribofilm formation to increase with load. The contact voltage response revealed the formation of a nonconductive tribofilm regardless of the applied load (Figure 4b). While the steady-state contact voltage was not sensitive to the load, both the incubation and transient stages of the contact voltage increased with the load. For *L* < 10.15 kg, tribofilm formation was instigated soon after the start of sliding and subsequently progressed fairly rapidly, consistent with the findings of another study [35]. However, a bimodal contact voltage response was found for *L* = 10.15 kg, suggesting that the critical load for blend 2 was close to 10.15 kg. About half of the tests performed with the highest load demonstrated tribofilm formation after relatively long incubation times and long transient stages (i.e., mode M2), implying prolonged duration of metal-to-metal contact and low rate of tribofilm formation, respectively. These load-dependent effects on tribofilm formation indicate a higher wear rate during the initial stage of sliding under the highest load.

**Figure 5:**
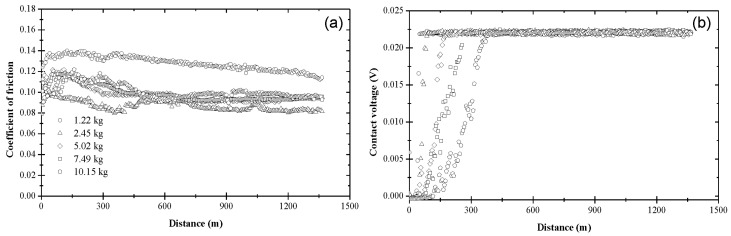
(**a**) Coefficient of friction and (**b**) contact voltage versus sliding distance and load for blend 3 (base oil + 0.08 wt% ZDDP).

Figure 5 shows that the coefficient of friction and contact voltage due to blend 3 exhibited a similar load dependence and variation with sliding distance with those attributed to blend 2. The resemblance between blends 2 and 3 can be explained by considering the chemical composition of these blends. The main difference is that blend 3 contained slightly more ZDDP (0.08 wt%) than blend 2 (0.05 wt%). However, despite the similar trends, the tribofilm produced by blend 3 yielded a steady-state coefficient of friction in the range of 0.08–0.10, which was lower than that of the tribofilm produced by blend 2, except for the lightest load, presumably due to compositional differences of the tribofilms formed under different loads. Similar to blend 2, the contact voltage response due to blend 3 revealed the formation of a nonconductive tribofilm at all loads (Figure 5b). Again, the incubation and transition stages increased with the applied load. However, the duration of these stages was shorter and a bimodal behavior of the contact voltage did not occur in the experiments performed with the highest load (10.15 kg), i.e., a nonzero steady-state contact voltage response (mode M2) evolved in all tests performed with a load equal to 10.15 kg. Since a zero steady-state contact voltage was not found in any of these experiments, the critical load for blend 3 was predicted to be above 10.15 kg. A comparison of the results shown in Figure 4 and Figure 5 shows that the tribofilm due to blend 3 displayed better friction properties than the tribofilm formed from blend 2, especially under high load, apparently due to the higher ZDDP concentration in blend 3, which enhanced the process of tribofilm formation.

**Figure 6:**
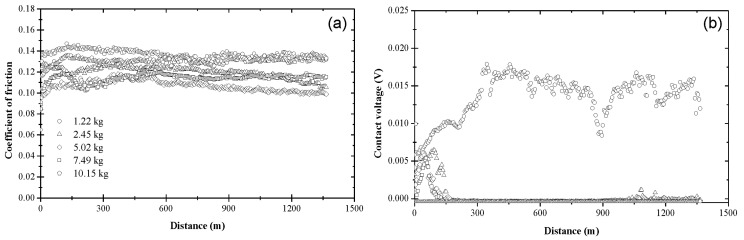
(**a**) Coefficient of friction and (**b**) contact voltage versus sliding distance and load for blend 4 (base oil + 0.1 wt% dispersant A).

Figure 6 shows the coefficient of friction and contact voltage as functions of sliding distance and load due to blend 4. In contrast to blends 2 and 3, the friction behavior due to blend 4 showed a similar trend with that due to blend 1 (Figure 3a), demonstrating a subtler running-in period. The coefficient of friction displayed small variations in the range of 0.10–0.14 with increasing sliding distance (Figure 6a). The highest friction coefficients were obtained with the lightest load, presumably due to the more significant effect of surface roughness under light-load sliding conditions. The evolution of the contact voltage with sliding distance (Figure 6b) did not reveal the formation of a protective tribofilm. Despite the nonzero contact voltage during the first 150 m of sliding, the steady-state response indicated that a nonconductive tribofilm did not form in the presence of blend 4. This was expected since the ethylene carbonate-treated bis-succinimide dispersant A is not known to react with steel to form an antiwear tribofilm, but to maintain the sliding track clean by dispersing the wear debris. The erratic contact voltage response obtained with the lightest load may be attributed to the combined effects of the native oxide film and the fine wear debris trapped at the contact interface, which were secondary effects under higher loads due to the removal of the oxide film and the wear debris by the intensified normal and shear surface tractions. As anticipated, the results shown in Figure 6 indicate that blending base oil with bis-succinimide dispersant alone cannot enhance the tribological properties.

**Figure 7:**
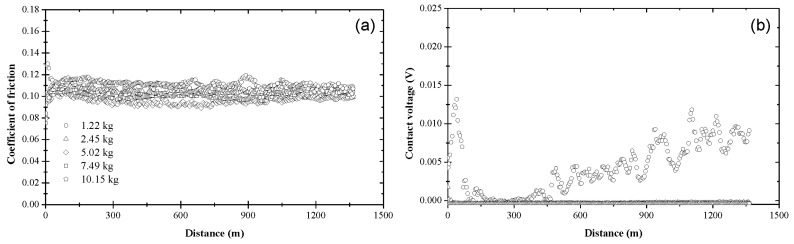
(**a**) Coefficient of friction and (**b**) contact voltage versus sliding distance and load for blend 5 (base oil + 0.1 wt% dispersant B).

The coefficient of friction and contact voltage versus sliding distance and load due to blend 5 shown in Figure 7 reveal similar trends with those associated with blend 4. The only difference between blends 4 and 5 is the dispersant. Blend 5 contained a high molecular weight dispersant of higher dispersancy and better capability to suspend the contaminants in the oil during testing. The coefficient of friction due to blend 5 varied in the range of 0.10–0.12 (Figure 7a), i.e., less than that due to blend 4 (Figure 6a). Therefore, it may be inferred that the tribofilm produced by blend 5 demonstrated better friction characteristics than that produced by blend 4. However, similar to blend 4, the evolution of the contact voltage did not show tribofilm formation in the presence of blend 5 at any load (Figure 7b). The erratic contact voltage response obtained with the lightest load can be attributed to the effect of surface roughness and the fine wear debris trapped at the contact interface under light-load sliding conditions. The lower contact voltage measured in the presence of blend 5 at a light load compared to blend 4 may be associated with dispersancy differences. It was presumed that blend 5 produced more intimate metal-to-metal contact due to its higher dispersancy, which reduced the amount of wear debris on the sliding track. This decreased the contact voltage, especially for loads above 1.22 kg where the resulting higher surface tractions were effective in pushing the wear debris out of the contact interface.

**Figure 8:**
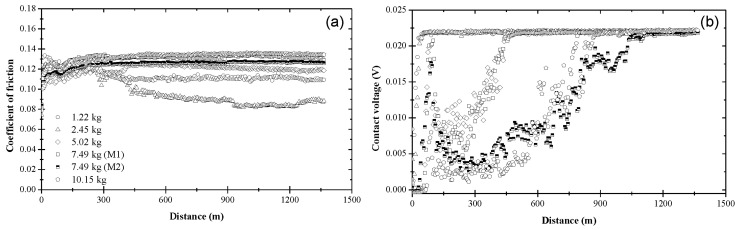
(**a**) Coefficient of friction and (**b**) contact voltage versus sliding distance and load for blend 6 (base oil + 0.05 wt% ZDDP + 0.1 wt% dispersant A).

The variation of the coefficient of friction with sliding distance due to blend 6 demonstrated a load dependence (Figure 8a). The coefficient of friction was initially in the range of 0.10–0.13, reaching a steady state in the range of 0.08–0.13, depending on load. For lighter loads (1.22 and 2.45 kg), the coefficient of friction exhibited a similar trend with that observed with blend 2 (Figure 4a), while for heavier loads (7.49 and 10.15 kg) the friction coefficient response resembled those due to blends 1, 4, and 5 (Figure 3a, Figure 6a, and Figure 7a, respectively). The different trends in Figure 8a suggested that the surface tractions affected the formation and composition of the produced tribofilms. Blend 6 yielded a complex contact voltage behavior, particularly at high loads, probably due to the competing effects of ZDDP and dispersant A. Although all contact voltage responses indicated the development of a nonconductive tribofilm (Figure 8b), the pathway of tribofilm formation demonstrated a load dependence. Light loads produced a short transient stage, suggesting a fairly rapid process of tribofilm formation similar to that due to blend 2 (Figure 4b) and blend 3 (Figure 5b). Increasing the load above 2.45 kg prolonged the transient stage and produced vigorous contact voltage fluctuations. A two-stage process was observed for *L* ≥ 5.02 kg. The sharp increase of the contact voltage during the first stage designated rapid tribofilm formation, as if ZDDP was the predominant driving force. However, the subsequent erratic contact voltage behavior suggested that the contact conditions were not conducive to the formation of a stable tribofilm. This was followed by a second stage characterized by a slower rise of the contact voltage (i.e., lower rate of tribofilm formation) than that seen in the first stage and a more pronounced load effect. This two-stage process implied that dispersants can become more active at high loads, interfering with tribofilm formation either by preventing the ZDDP molecules to access the sliding surface or by competing with ZDDP molecules for surface adsorption sites. A bimodal contact voltage behavior was observed for an intermediate load (7.49 kg). The bimodal behavior due to blend 6 not only illuminated the complexity of tribochemical reactions at loads close to the critical load, but also revealed a pronounced interference of the dispersant in the process of tribofilm formation.

**Figure 9:**
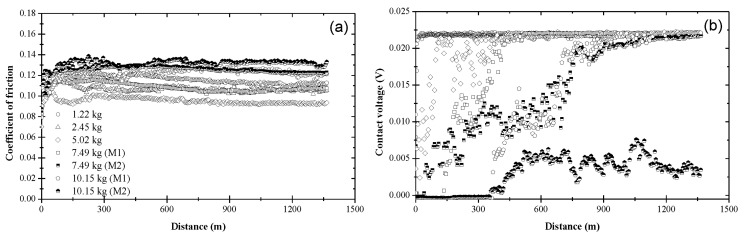
(**a**) Coefficient of friction and (**b**) contact voltage versus sliding distance and load for blend 7 (base oil + 0.05 wt% ZDDP + 0.1 wt% dispersant B).

Blend 7 had a similar composition with that due to blend 6 (Table 1) but contained a dispersant characterized by a higher molecular weight and better dispersancy. The coefficient of friction due to blend 7 was initially in the range of 0.08–0.12, stabilizing fairly soon in the range of 0.09–0.13 at steady state (Figure 9a). Contrary to blend 6, the friction behavior due to blend 7 did not demonstrate a clear load dependence. The lowest and highest coefficients of friction were obtained under sliding conditions of intermediate load (5.02 kg) and low (1.22 kg) or high (10.15 kg) loads, respectively. Blend 7 demonstrated even more complex pathways of tribofilm formation than blend 6, especially at high loads. In contrast to the friction behavior, the contact voltage response due to blend 7 revealed a load dependence (Figure 9b). For *L* ≤ 5.02 kg, tribofilm formation stabilized after a relatively short transient stage similar to that due to blend 3 (Figure 5b), but longer than that due to blend 6 (Figure 8b) for the same load. Significant differences in the contact voltage responses due to blends 6 and 7 were encountered at higher loads. In addition to a significantly extended transient stage, blend 7 yielded a bimodal behavior for loads equal to 7.49 and 10.15 kg and a mode M2 contact voltage response for a 0.15 kg load, indicating the formation of an unstable tribofilm. From the perspective of durable (stable) tribofilm formation, the results shown in Figure 8 and Figure 9 indicate a superior performance for blend 6 despite the better dispersant incorporated in blend 7. Therefore, it appears that the higher dispersancy of dispersant B was detrimental to the efficacy of ZDDP to form a durable tribofilm. This finding is illustrative of the competition between ZDDP and dispersant molecules to access and decompose at the contact interface in the presence of surface tractions, which is a critical factor for tribofilm formation.

### 3.2. Steady-State Coefficient of Friction

**Figure 10:**
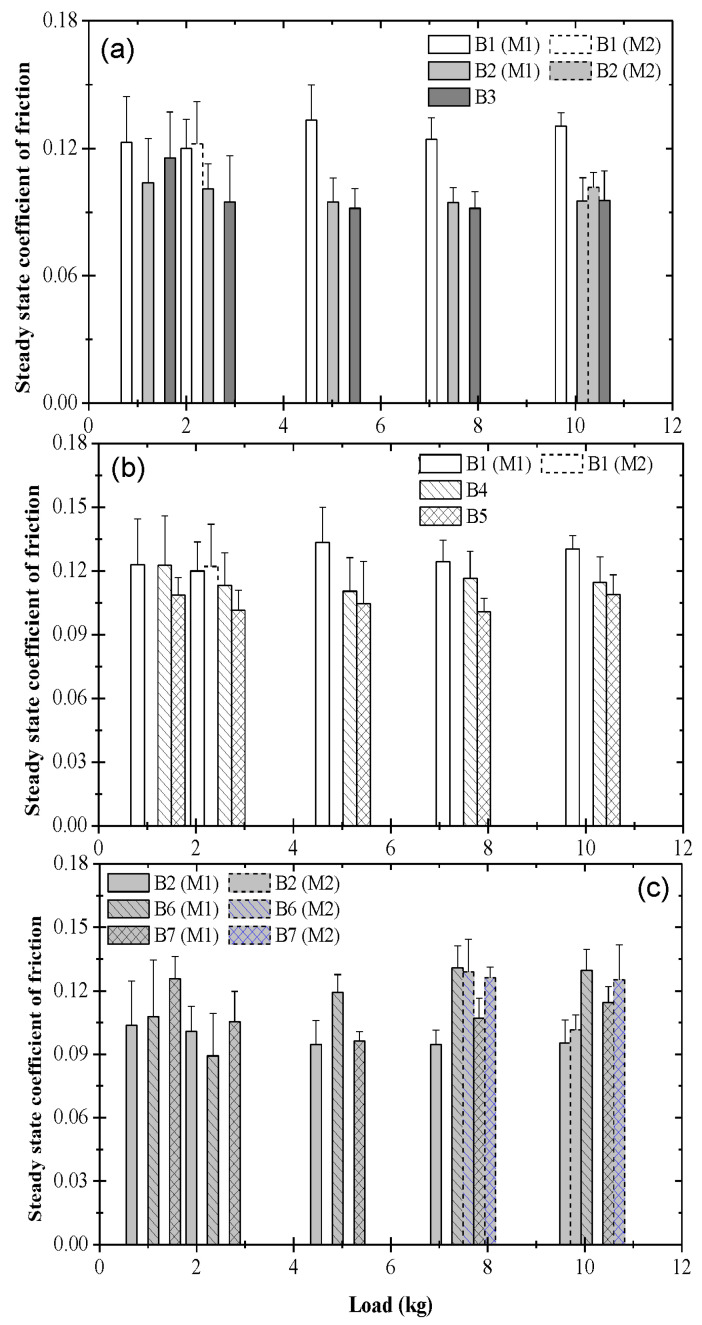
Steady-state coefficient of friction versus load for different blends: (**a**) B1 = blend 1 (base oil), B2 = blend 2 (base oil + 0.05 wt% ZDDP), and B3 = blend 3 (base oil + 0.08 wt% ZDDP); (**b**) B1 = blend 1 (base oil), B4 = blend 4 (base oil + 0.1 wt% dispersant A), and B5 = blend 5 (base oil + 0.1 wt% dispersant B); (**c**) B2 = blend 2 (base oil + 0.05 wt% ZDDP), B6 = blend 6 (base oil + 0.05 wt% ZDDP + 0.1 wt% dispersant A), and B7 = blend 7 (base oil + 0.05 wt% ZDDP + 0.1 wt% dispersant B). (M1 and M2 indicate bimodal behavior; error bars indicate one standard deviation above the mean value).

To provide further insight into the friction characteristics of the different tribofilms illuminated by the coefficient of friction and contact voltage measurements, the load effect on the steady-state coefficient of friction due to each blend was investigated. To better differentiate the effects of ZDDP and dispersants on the frictional characteristics, the comparison was made among the steady-state coefficients of friction versus load (Figure 10) for blends assigned to the following three groups: base oil with and without different concentrations of ZDDP (blends 1, 2, and 3) (Figure 10a), base oil with dispersant A or B (blends 1, 4, and 5) (Figure 10b), and base oil containing 0.05 wt% ZDDP with or without dispersant A or B (blends 2, 6, and 7) (Figure 10c). Figure 10a shows an insignificant load effect on the steady-state friction behavior associated with pure base oil and base oil containing either 0.05 or 0.08 wt% ZDDP. Although improved friction characteristics were obtained in the presence of ZDDP, especially at high loads, the effect of decreasing the ZDDP concentration from 0.08 to 0.05 wt% on the steady-state friction performance was minor. Similar to the former group of blends, the load effect on the steady-state friction characteristics due to pure base oil and base oil containing 0.1 wt% dispersant A or B was marginal. Although the general trend was for the coefficient of friction to decrease in the presence of dispersants, the decrease was not as pronounced as for the ZDDP-containing blends of the previous group. The slightly lower coefficient of friction due to blend 5 encountered throughout the load range may be attributed to the enhanced dispersancy of dispersant B compared to that of dispersant A incorporated in blend 4. Therefore, it may be inferred that dispersant B suspended more effectively the wear debris in the oil, reducing the plowing action of wear debris trapped at the contact interface during sliding. To distinguish the dispersant effect on the friction behavior, the steady-state coefficients of friction at different loads associated with base oil containing 0.05 wt% ZDDP with or without 0.1 wt% dispersant A or B are contrasted in Figure 10c. Although both the ZDDP and the dispersants reduced the coefficient of friction when mixed with base oil separately (Figure 10a,b), a comparison of the coefficient of friction data due to blends 6 and 7 with those due to blend 2 indicates that, in general, a higher steady-state coefficient of friction was obtained when ZDDP and dispersant coexisted in a blend. Moreover, unlike the comparison between blends 4 and 5 (Figure 10b), it was more difficult to determine whether blend 6 acted as a better friction modifier than blend 7 or vice versa, because blend 6 yielded lower coefficients of friction at light loads, whereas blend 7 was more effective in reducing friction at high loads. These results indicate that although better dispersancy may reduce friction when the dispersant is the only additive, this effect may be diminished when the dispersant is mixed in a blend together with other competing chemicals.

### 3.3. Critical Distance for Stable Tribofilm Formation

**Figure 11:**
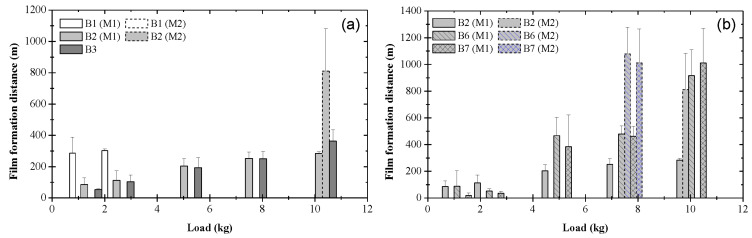
Film formation distance as a function of load for different blends: (**a**) B1 = blend 1 (base oil), B2 = blend 2 (base oil + 0.05 wt% ZDDP), and B3 = blend 3 (base oil + 0.08 wt% ZDDP); (**b**) B2 = blend 2 (base oil + 0.05 wt% ZDDP), B6 = blend 6 (base oil + 0.05 wt% ZDDP + 0.1 wt% dispersant A), and B7 = blend 7 (base oil + 0.05 wt% ZDDP + 0.1 wt% dispersant B). (M1 and M2 indicate bimodal behavior; error bars indicate one standard deviation above the mean value).

Although the coefficient of friction is one of many ways to evaluate the friction properties associated with a particular blend, a more subtle indicator of the friction characteristics of the formed tribofilms is the critical distance for steady-state tribofilm formation *d_ss_* (Figure 2). A comparison of *d_ss_* versus load due to different blends is shown in Figure 11. A zero distance for tribofilm formation implies test conditions not conducive to the formation of a stable, nonconductive tribofilm, as demonstrated by the contact voltage measurements. Figure 11a shows the distance for tribofilm formation as a function of load for pure base oil and base oil containing either 0.05 or 0.08 wt% ZDDP. In the presence of pure base oil, a sustainable tribofilm (probably an oxide film) formed only under light loads. However, in the presence of ZDDP, a stable tribofilm formed at all loads. The data for the blends containing ZDDP reveal a fairly linear correlation between tribofilm formation distance and applied load. Although the blends containing ZDDP produced a similar critical distance for tribofilm formation, it appears that the higher concentration of ZDDP in blend 3 promoted a faster formation of a sustainable tribofilm. The rapid formation of a stable tribofilm and low friction observed with blends 2 and 3 are illustrative of the efficacy of ZDDP to reduce friction and to effectively protect the sliding surfaces. Data of the critical distance for tribofilm formation corresponding to the blends of the second group (i.e., base oil with dispersant A or B (blends 1, 4, and 5)) are not presented here because the contact voltage responses due to blends 4 and 5 did not provide evidence of tribofilm formation at any load. Dispersants reduce friction by inhibiting wear debris to remain at the contact interface and scratch the surfaces. Thus, the incorporation of a dispersant in the base oil did not lead to tribofilm formation even at light loads. Figure 11b shows a comparison of the critical distance for tribofilm formation versus load for base oil containing 0.05 wt% ZDDP with or without 0.1 wt% dispersant A or B (i.e., blends 2, 6, and 7). While the dispersant promoted tribofilm formation at light loads, it produced an opposite effect at high loads. Unlike the blends consisting of base oil and ZDDP, the critical distance for stable tribofilm formation did not show a linear load dependence when both ZDDP and dispersant were incorporated in the base oil. The critical distance for stable tribofilm formation increased with the applied load almost exponentially. Despite the better dispersancy of dispersant B, tribofilm formation did not always occur sooner in the presence of blend 7, especially at high loads.

The presented results and analysis of the temporal variation of the coefficient of friction and contact voltage exhibited by the tribofilms generated by different blends for a range of loads illustrate the efficacy of the present experimental approach to discern the dynamics and critical load conditions for stable tribofilm formation and to demonstrably determine the tribofilm yielding the best friction behavior. The chemical composition, thickness, and wear mechanisms of the tribofilms produced by the blends examined in this study will be presented in a forthcoming publication. Future work may focus on the establishment of correlations between in situ friction and contact voltage measurements and ex situ characterization of the wear mechanisms of tribofilms produced by different oil formulations.

## 4. Conclusions

The complex nature of tribofilm formation on steel surfaces boundary lubricated with different blends was investigated in the light of in situ measurements of the coefficient of friction and contact voltage. The transient and steady-state coefficient of friction, contact voltage, and critical sliding distance (time) for stable tribofilm formation were used to evaluate the efficacy of tribofilms formed at elevated temperature under various loads. A bimodal contact voltage behavior was generally observed for loads close to the critical load of each tribofilm, implying that a slight disturbance in the test environment could positively or negatively affect the formation of a stable and durable tribofilm. The critical sliding distance for the formation of a stable tribofilm demonstrated a linear load dependence for blends consisting of base oil and ZDDP and an exponential load dependence for blends comprising base oil and a mixture of ZDDP and dispersant. Although the addition of ZDDP or dispersant in the base oil reduced the steady-state coefficient of friction obtained with pure base oil, the coefficient of friction increased when ZDDP was blended together with a dispersant into the base oil. Improved dispersancy reduced the coefficient of friction in the presence of a dispersant, whereas it increased the coefficient of friction when the dispersant was used together with ZDDP, indicating a competition between ZDDP and dispersant molecules to access and decompose at the contact interface. In the absence of ZDDP, a protective tribofilm formed only under light loads in the presence of pure base oil, whereas tribofilm formation was not observed in the presence of blends consisting of base oil and dispersant. The tribofilm exhibiting the best friction behavior was produced by the blend containing base oil, a reduced amount of ZDDP, and a bis-succinimide dispersant treated with ethylene carbonate. This study demonstrated that the combination of in situ measurements of the coefficient of friction and contact voltage represents an effective experimental approach for capturing the dynamics of tribofilm formation resulting from various lubricant formulations under elevated-temperature boundary lubrication conditions.

## Figures and Tables

**Table 1:** Chemical composition of oil blends.

Blend	Composition	Concentration (ppm)			
		N	P	Zn	S
1	base oil	–	–	–	19
2	base oil + 0.05 wt% ZDDP	1.7	513	545	1042
3	base oil + 0.08 wt% ZDDP	–	803	868	1615
4	base oil + 0.1 wt% dispersant A	894	–	–	57
5	base oil + 0.1 wt% dispersant B	175	–	–	26.7
6	base oil + 0.05 wt% ZDDP + 0.1 wt% dispersant A	888	516	552	1102
7	base oil + 0.05 wt% ZDDP + 0.1 wt% dispersant B	175	519	551	1081

**Table 2:** Mechanical properties of as-received ball and disk specimens.

Property	Specimen	
	Ball	Disk
Yield strength (MPa)	560	560
Rockwell C hardness	62 ± 3	60 ± 3
Ultimate tensile strength (MPa)	700	700
Elastic modulus (GPa)	193	193
Shear modulus (GPa)	66.5	66.5
Poisson’s ratio	0.30–0.31	0.30–0.31
Elongation in 5 cm (%)	25	25
Reduction in area (%)	57	57
Root-mean-square roughness (µm)	<50.8	(25.4–50.8) *
Diameter (cm)	0.8	3.2
Thickness (cm)	n/a	1

* The disk specimens were polished to an average roughness of 21.1 ± 7.9 nm.

## Data Availability

Data are contained within the article and Supplementary Materials.

## Supplementary Materials

The following supporting information can be downloaded at: https://www.mdpi.com/article/10.3390/ma17061335/s1.
