# Supplementary material for: Dynamics of Tribofilm Formation in Boundary Lubrication Investigated Using In Situ Measurements of the Friction Force and Contact Voltage
Source: Materials (Basel). 2024 Mar 14;17(6):1335. doi: 10.3390/ma17061335 (PMC10972069; doi:10.3390/ma17061335)
Supplement: Supplementary file 1 [file materials-17-01335-s001.zip › materials-2899386-supplementary.pdf]

Supplementary Material

I. Coefficient of friction and contact voltage

Statistical results of the coefficient of friction and contact voltage for various blends and loads are given below. Error bars indicate one standard deviation above and below each data point representing the average of five measurements obtained from 50 m sliding distance intervals.

Blend 1 (base oil)

Load = 1.22 kg

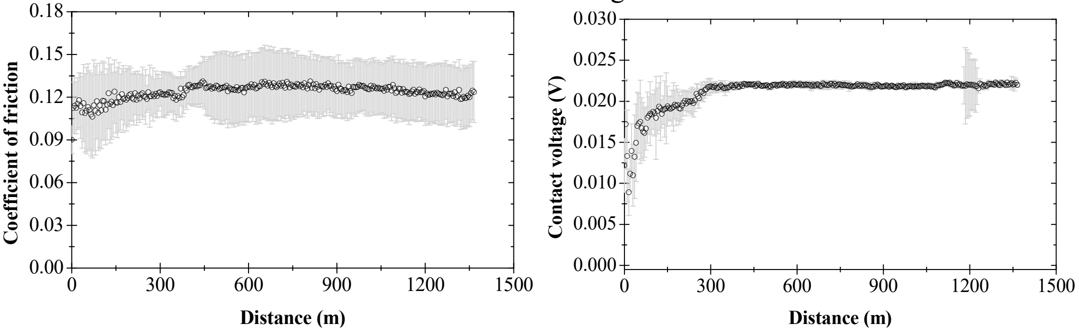

Load = 2.45 kg (mode M1)

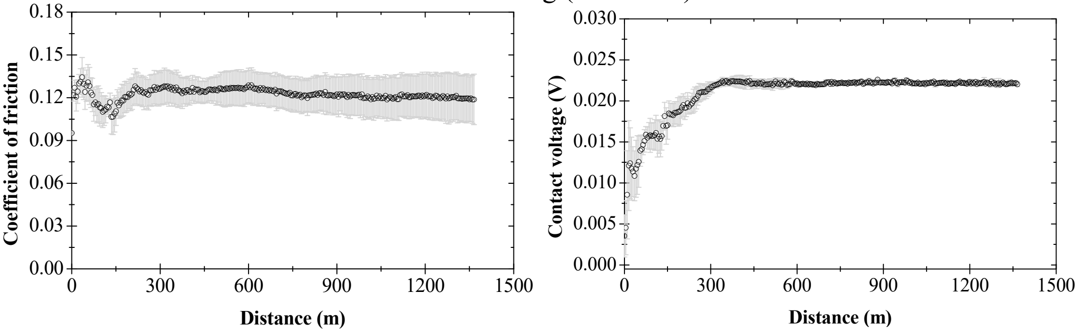

Load = 2.45 kg (mode M2)

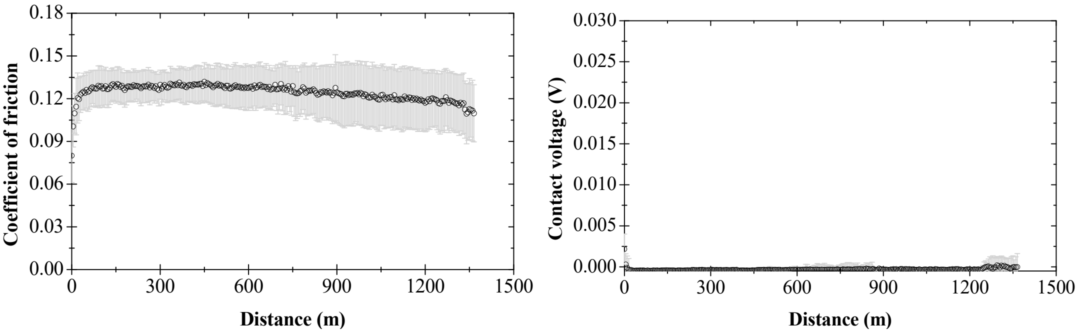

Load = 5.02 kg

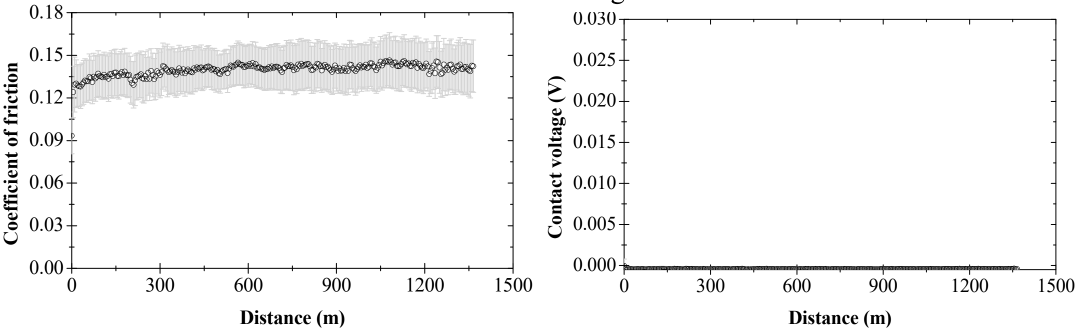

Load = 7.49 kg

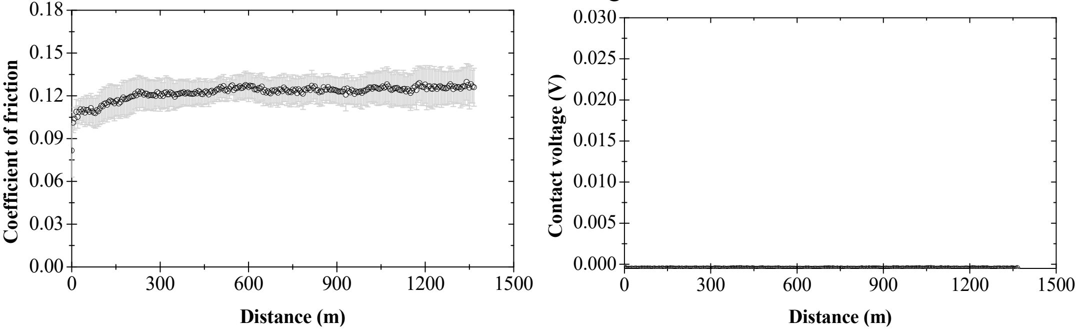

Load = 10.15 kg

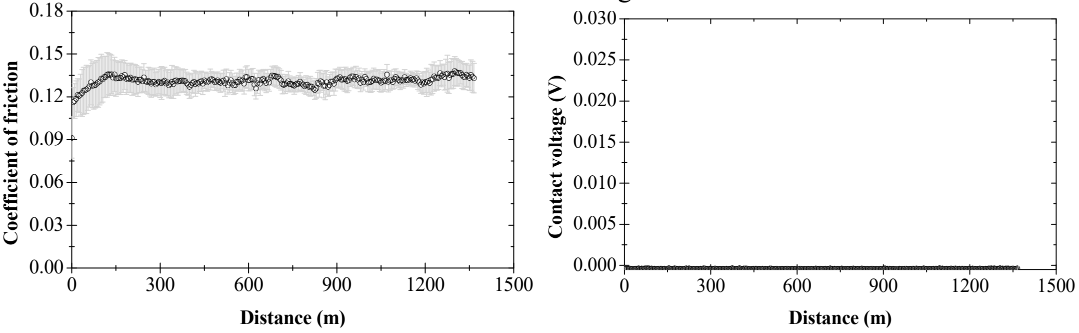

Blend 2 (base oil + 0.05% ZDDP)

Load = 1.22 kg

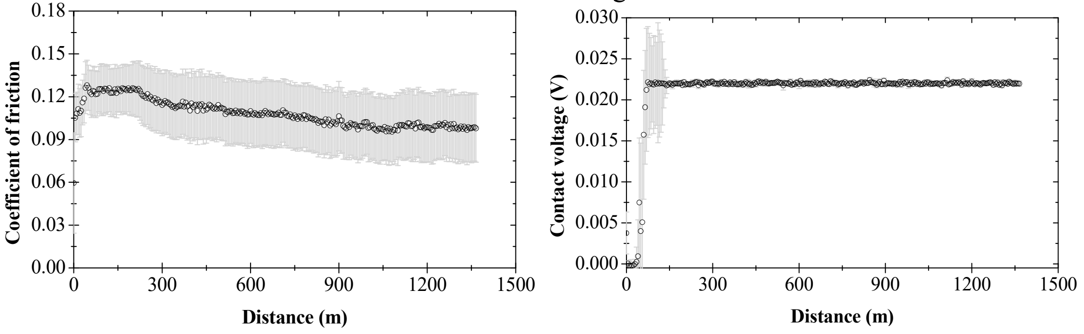

Load = 2.45 kg

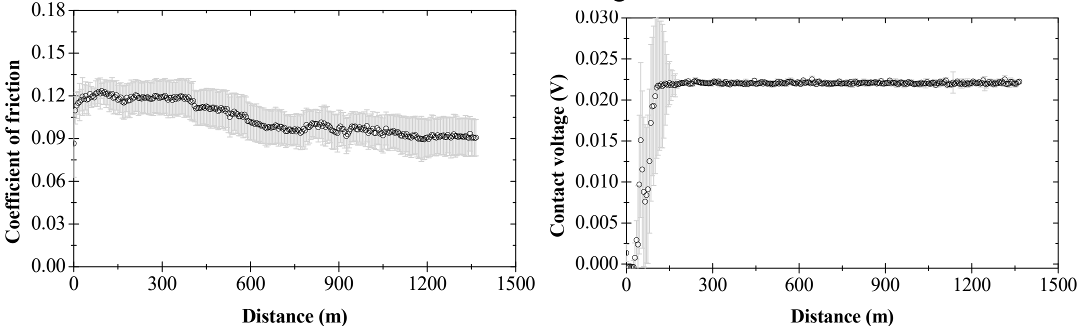

Load = 5.02 kg

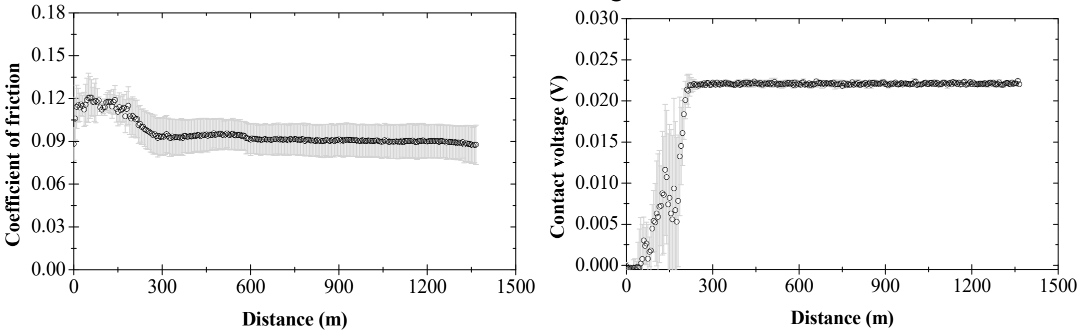

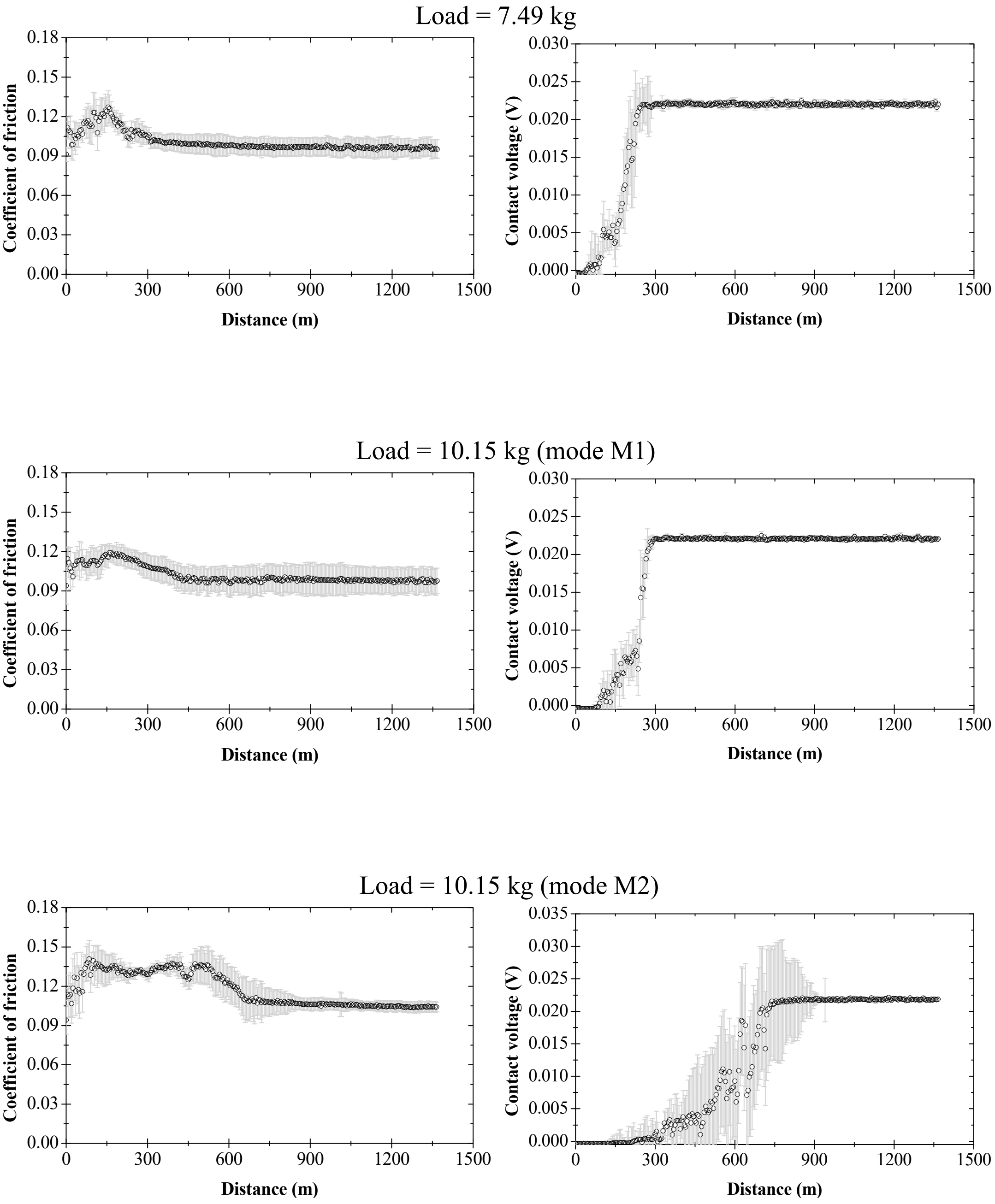
Load = 7.49 kg
Load = 10.15 kg (mode M1)
Load = 10.15 kg (mode M2)
Coefficient of friction
Contact voltage (V)
Distance (m)
0.00
0.03
0.06
0.09
0.12
0.15
0.18
0.000
0.005
0.010
0.015
0.020
0.025
0.030
0.035
0
300
600
900
1200
1500

Blend 3 (base oil + 0.08% ZDDP)

Load = 1.22 kg

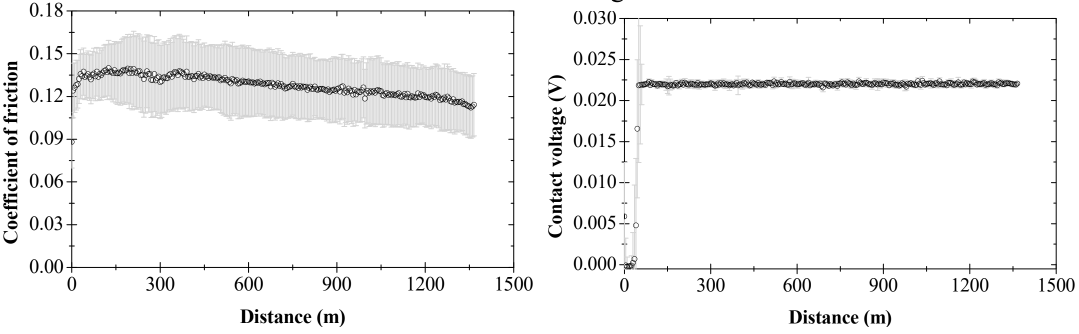

Load = 2.45 kg

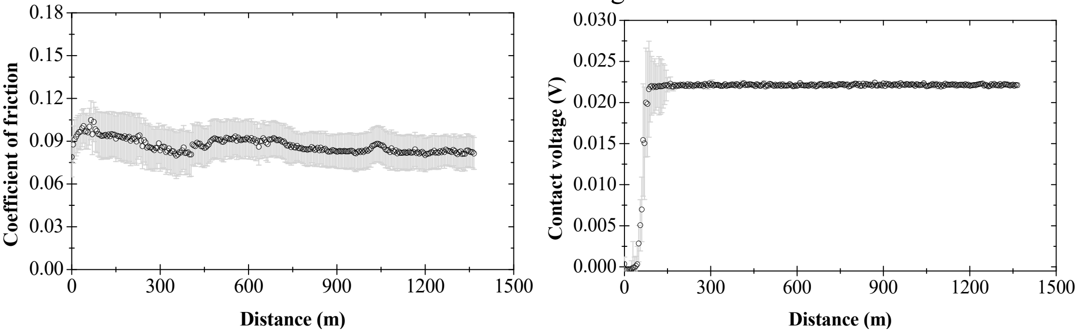

Load = 5.02 kg

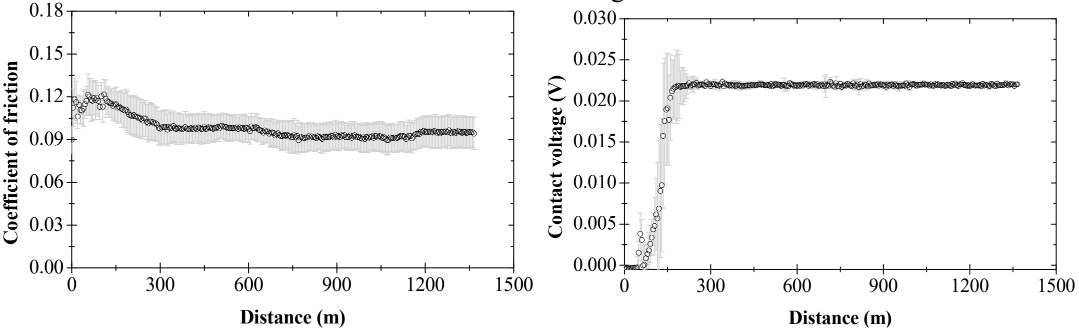

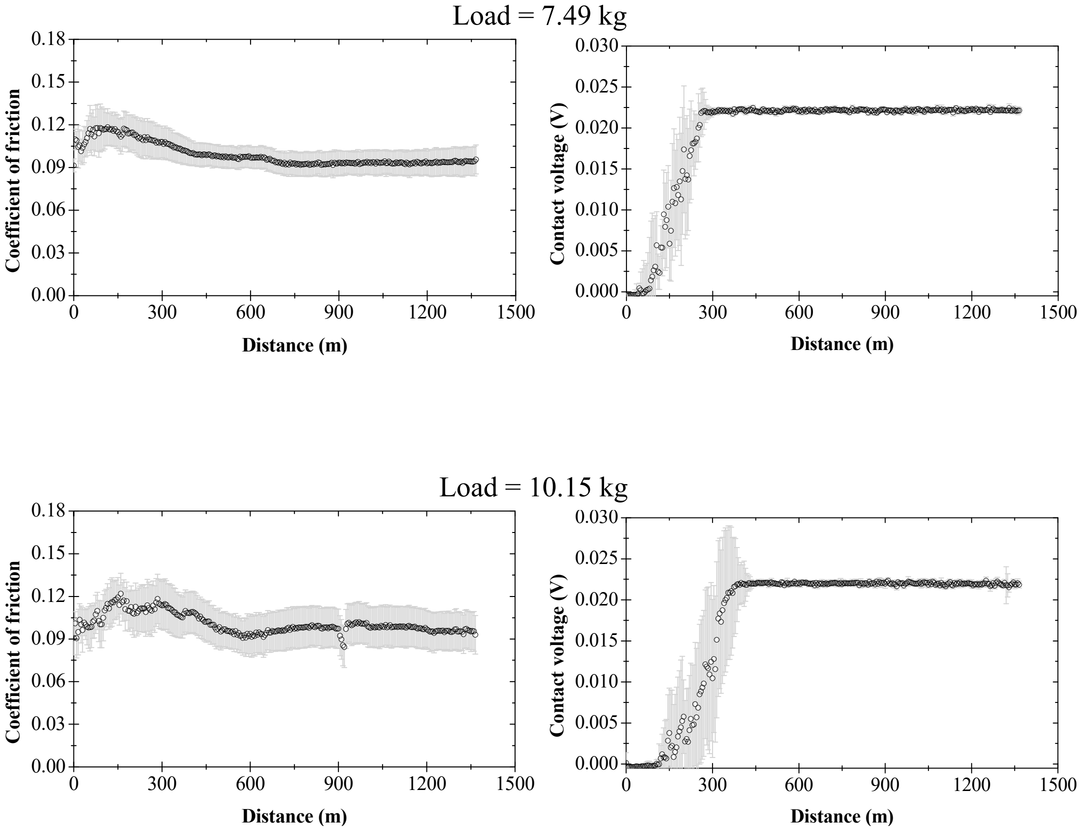
Load = 7.49 kg
Coefficient of friction
Distance (m)
Contact voltage (V)
Distance (m)
Load = 10.15 kg
Coefficient of friction
Distance (m)
Contact voltage (V)
Distance (m)

Blend 4 (base oil + 0.1% dispersant A)

Load = 1.22 kg

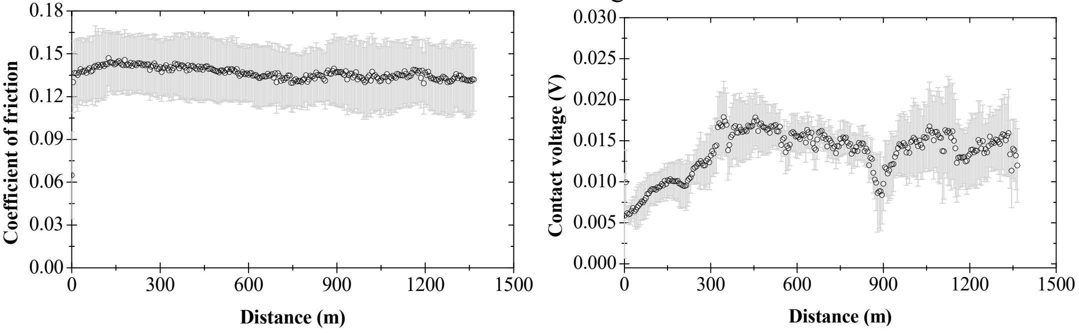

Load = 2.45 kg

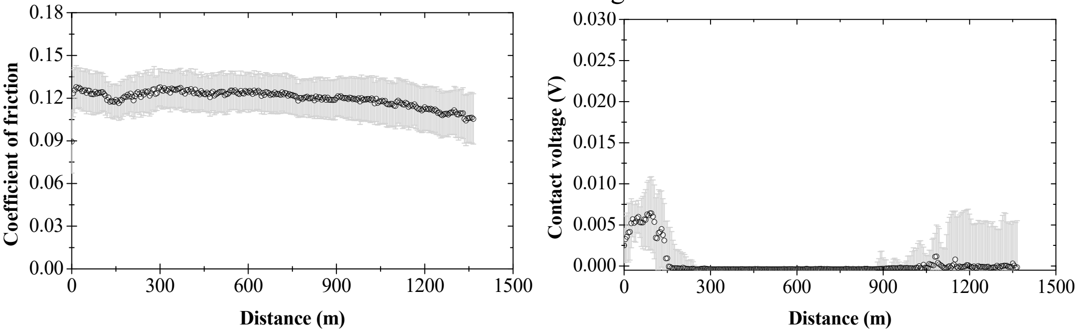

Load = 5.02 kg

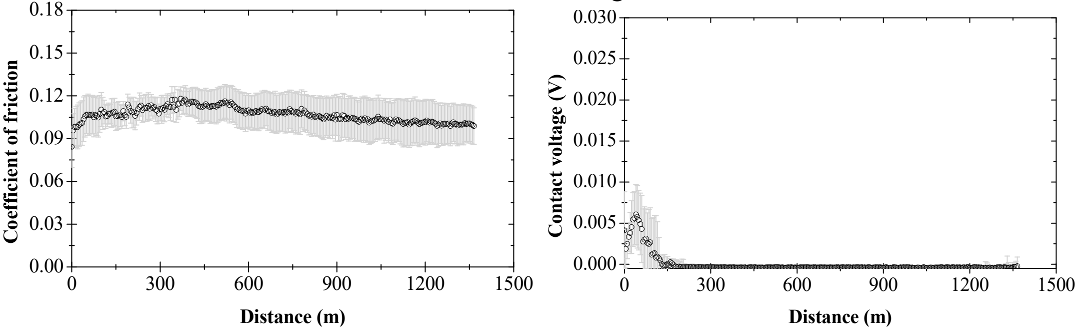

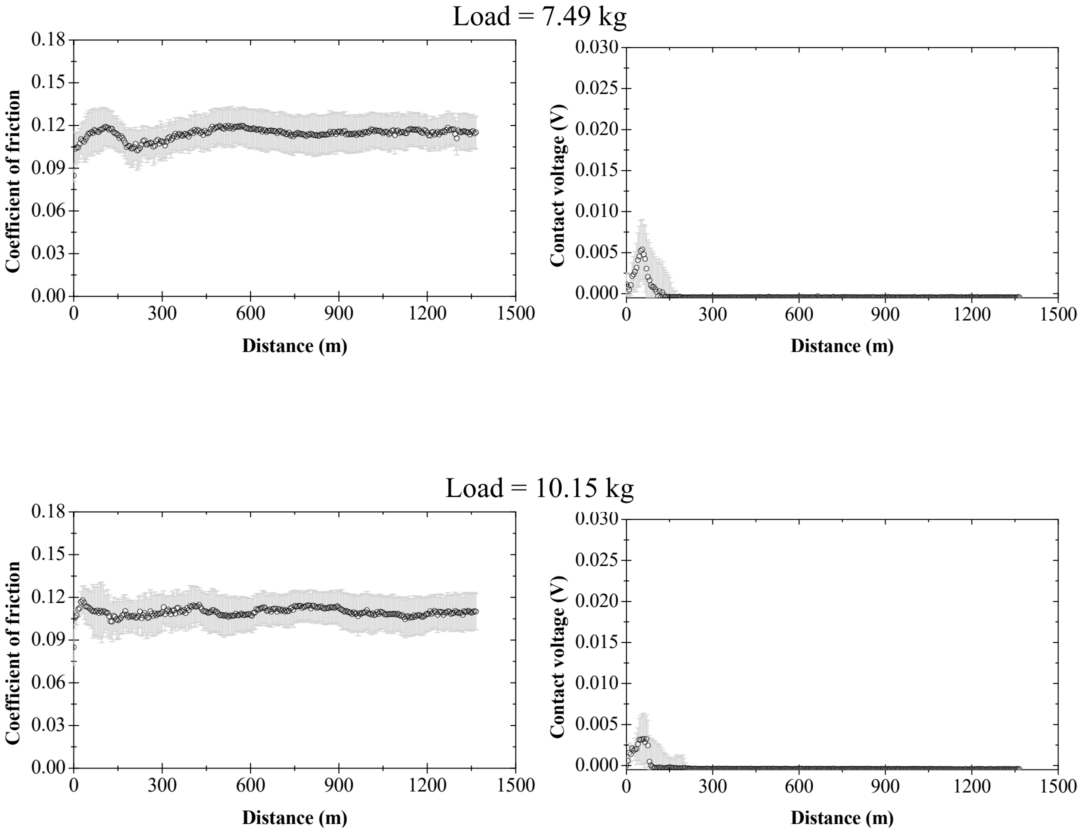
Load = 7.49 kg
Coefficient of friction
Contact voltage (V)
Distance (m)
Load = 10.15 kg
Coefficient of friction
Contact voltage (V)
Distance (m)

Blend 5 (base oil + 0.1% dispersant B)

Load = 1.22 kg

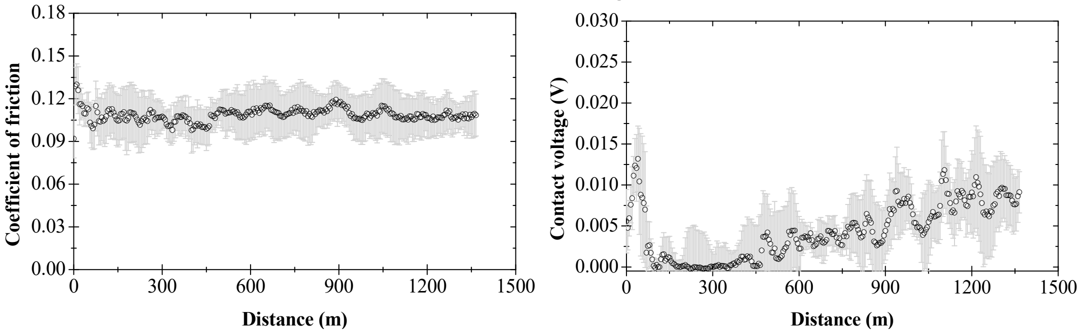

Load = 2.45 kg

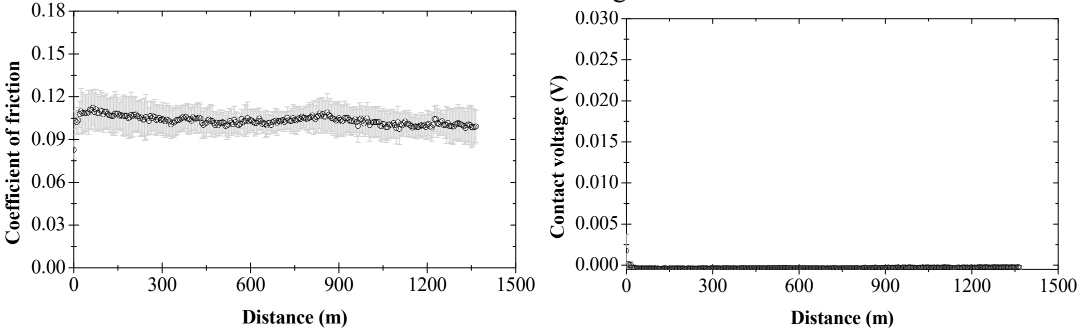

Load = 5.02 kg

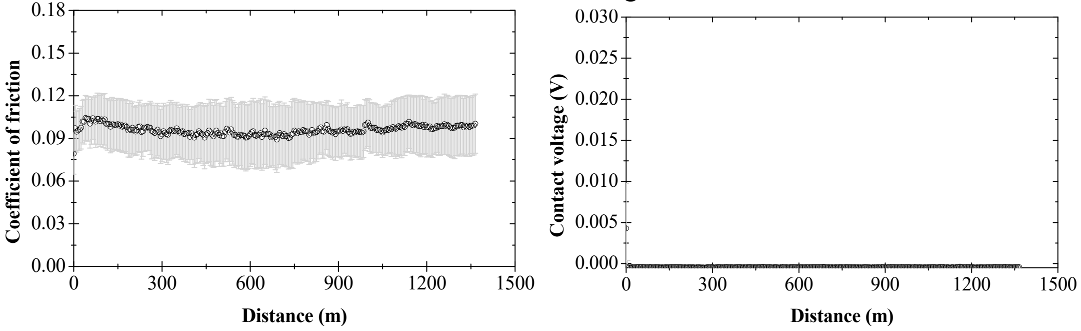

Load = 7.49 kg

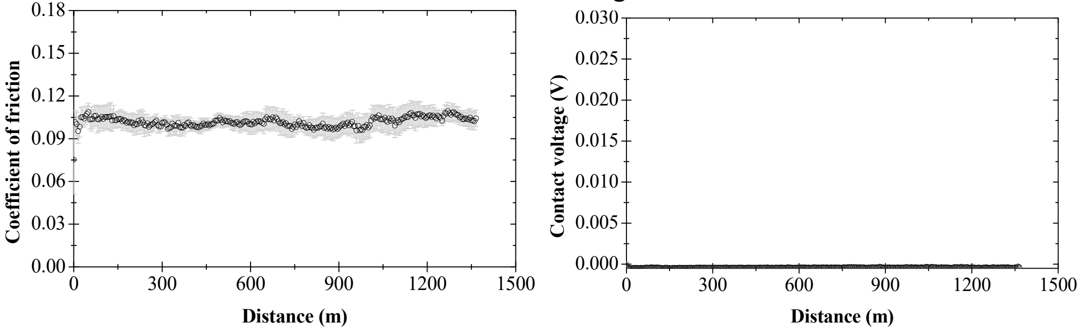

Load = 10.15 kg

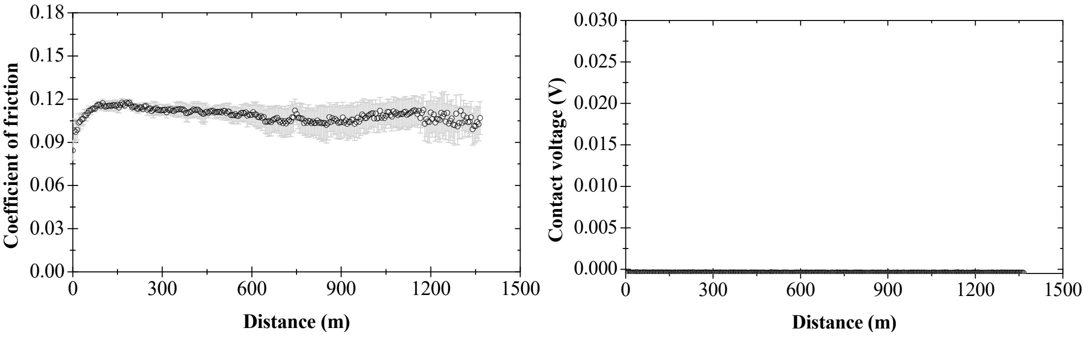

Blend 6 (base oil + 0.05% ZDDP + 0.1% dispersant A)

Load = 1.22 kg

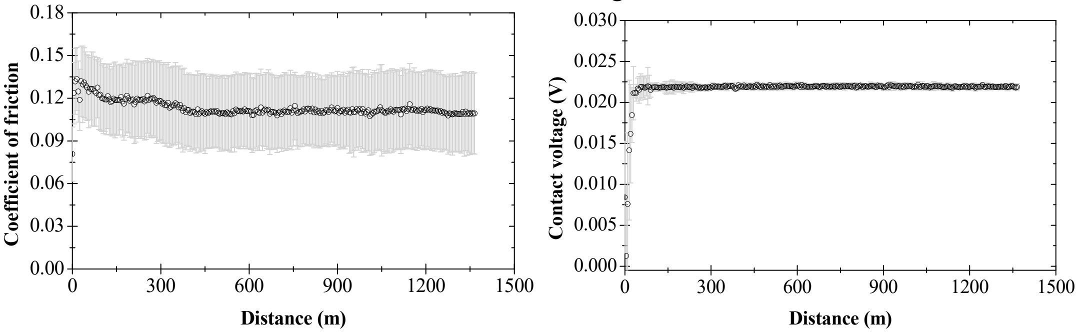

Load = 2.45 kg

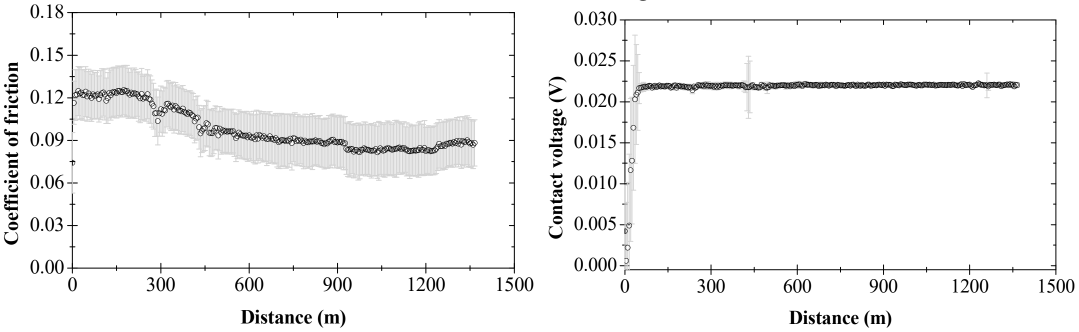

Load = 5.02 kg

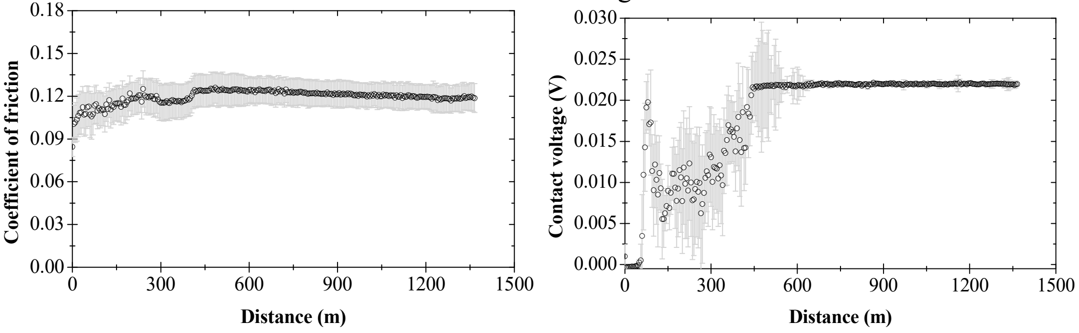

Load = 7.49 kg (mode M1)

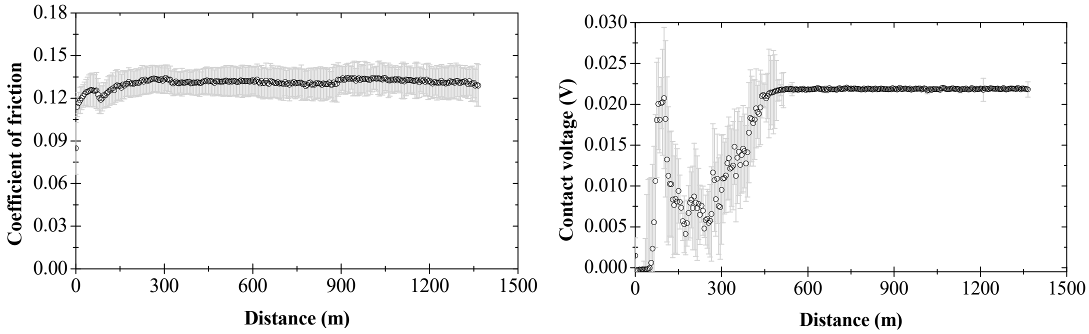

Load = 7.49 kg (mode M2)

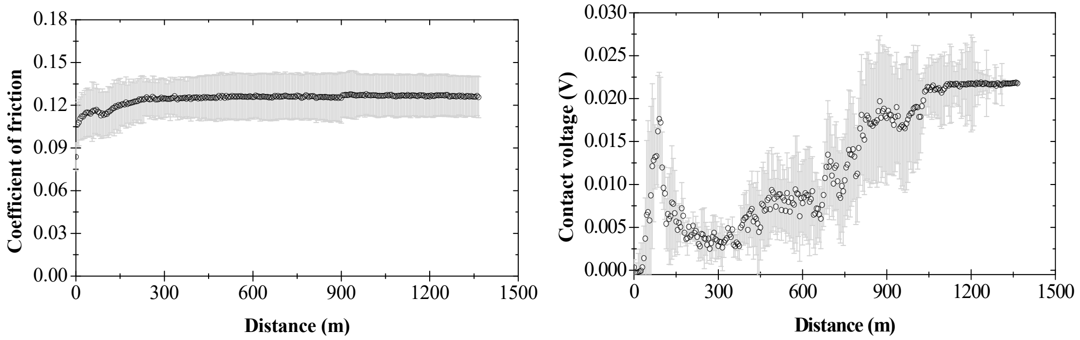

Load = 10.15 kg

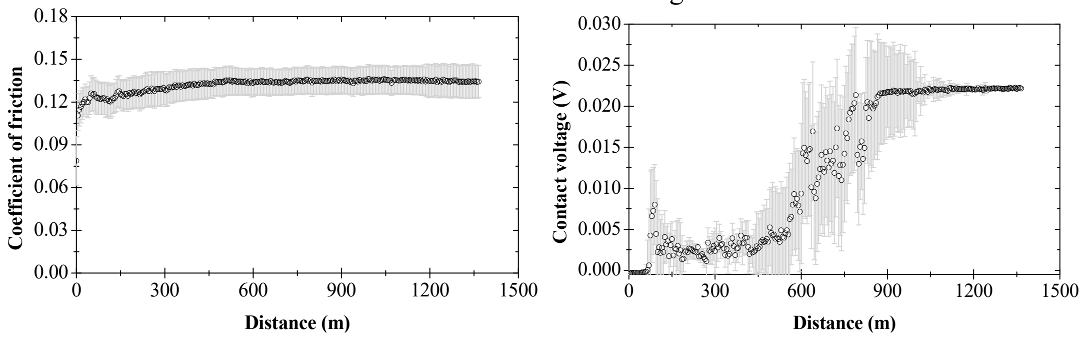

Blend 7 (base oil + 0.05% ZDDP + 0.1% dispersant B)

Load = 1.22 kg

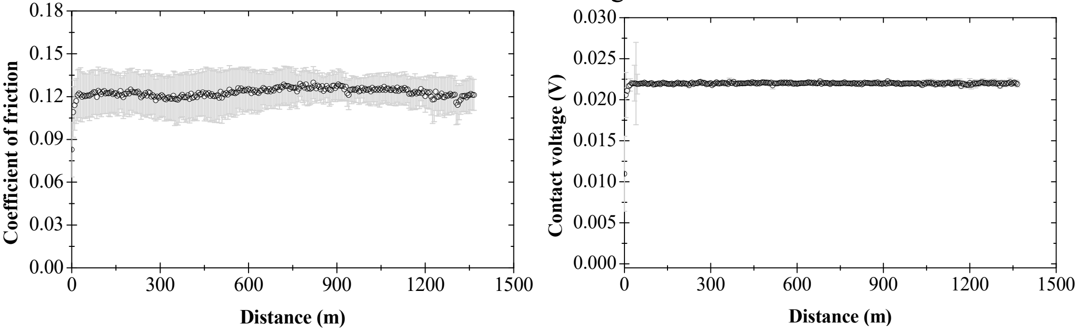

Load = 2.45 kg

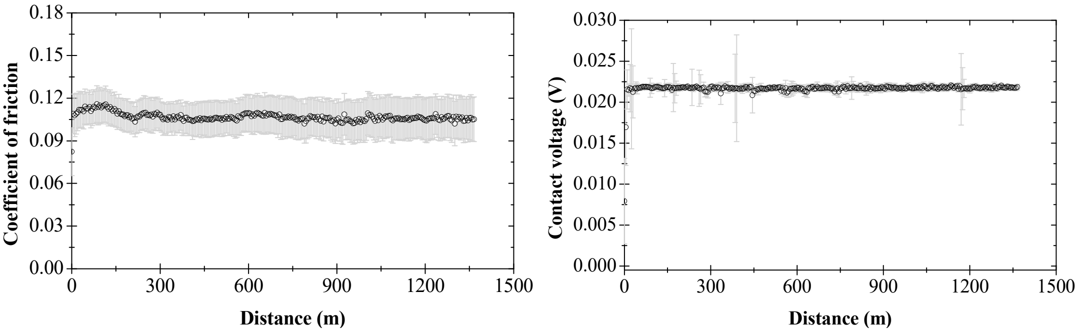

Load = 5.02 kg

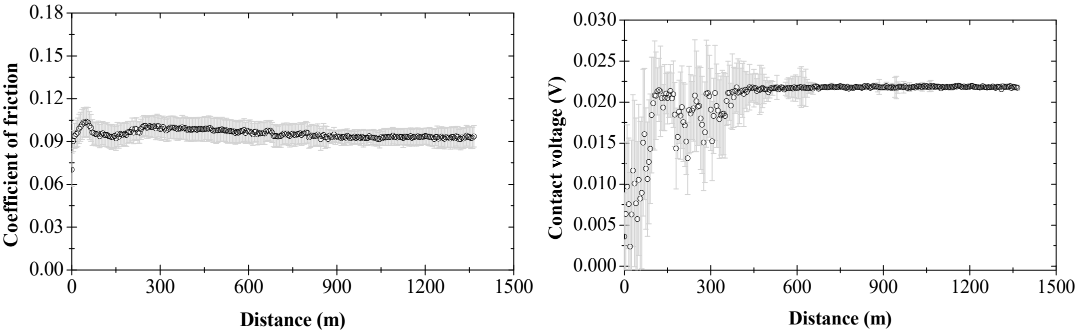

Load = 7.49 kg (mode M1)

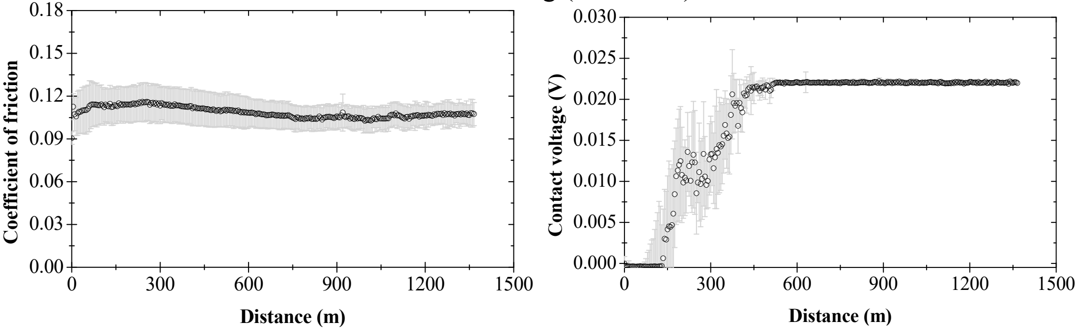

Load = 7.49 kg (mode M2)

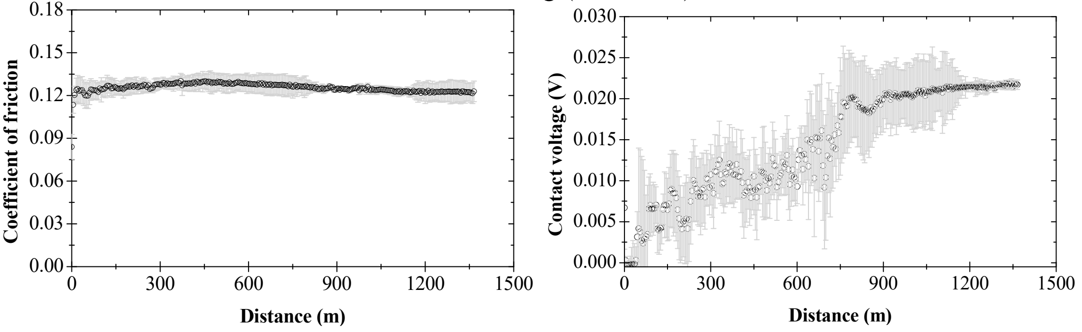

Load = 10.15 kg (mode M1)

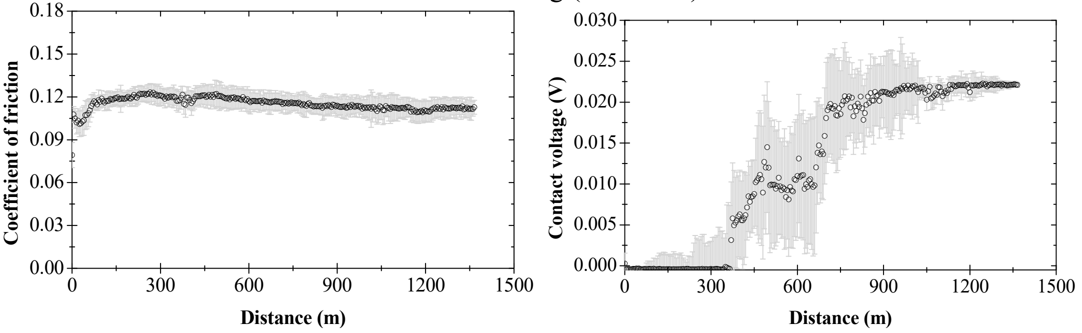

Load = 10.15 kg (mode M2)

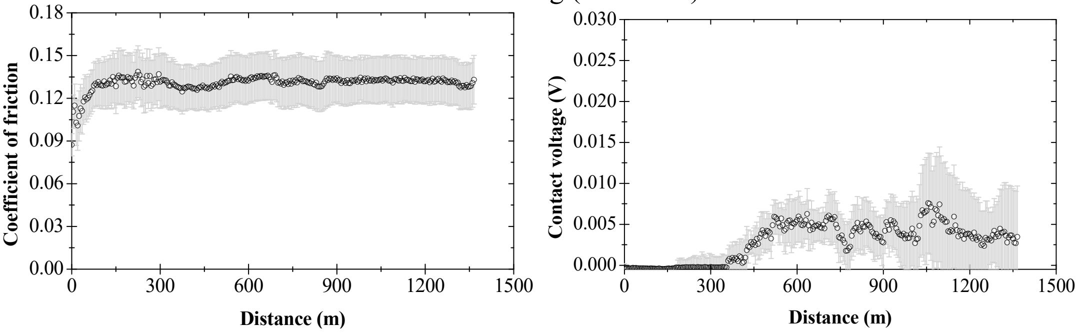

II. Characteristic distances of the evolution of tribofilm formation

Statistical results of the incubation, transient (transition), and film formation (steady state) distances for tribofilm formation by different blends at various loads derived from contact voltage measurements are given below.

	Load (kg)	Mode	Incubation Distance (m)			Transition Distance (m)			Film Formation Distance (m)		
Blend 1	1.22	--	6.0	±	6.5	281.0	±	98.8	287.0	±	101.5
	2.45	1	2.5	±	5.0	300.0	±	16.8	302.5	±	11.9
		2	>1365			Unknown			No Steady State Film		
	5.02	--	>1365			Unknown			No Steady State Film		
	7.49	--	>1365			Unknown			No Steady State Film		
	10.15	--	>1365			Unknown			No Steady State Film		
Blend 2	1.22	--	30.0	±	14.6	56.0	±	35.6	86.0	±	42.3
	2.45	--	32.0	±	11.5	81.0	±	51.6	113.0	±	60.6
	5.02	--	70.0	±	60.4	134.0	±	57.7	204.0	±	46.7
	7.49	--	57.0	±	28.2	196.0	±	48.3	253.0	±	41.3
	10.15	1	103.8	±	31.2	181.3	±	24.3	285.0	±	10.8
		2	264.0	±	113.9	499.0	±	266.0	763.0	±	237.8
Blend 3	1.22	--	32.0	±	11.0	21.0	±	8.2	53.0	±	6.7
	2.45	--	35.0	±	13.7	69.0	±	40.4	104.0	±	42.3
	5.02	--	59.0	±	23.3	135.0	±	73.9	194.0	±	64.2
	7.49	--	56.0	±	24.6	195.0	±	34.8	251.0	±	47.1
	10.15	--	80.0	±	32.0	283.0	±	96.0	363.0	±	71.5
Blend 4	1.22	--	1.0	±	2.2	Unknown			No Steady State Film		
	2.45	--	>1365			Unknown			No Steady State Film		
	5.02	--	>1365			Unknown			No Steady State Film		
	7.49	--	>1365			Unknown			No Steady State Film		
	10.15	--	>1365			Unknown			No Steady State Film		
Blend 5	1.22	--	224.0	±	204.9	Unknown			No Steady State Film		
	2.45	--	>1365			Unknown			No Steady State Film		
	5.02	--	>1365			Unknown			No Steady State Film		
	7.49	--	>1365			Unknown			No Steady State Film		
	10.15	--	>1365			Unknown			No Steady State Film		
Blend 6	1.22	--	2.0	±	2.7	86.0	±	117.1	88.0	±	116.0
	2.45	--	10.0	±	10.0	42.0	±	12.0	52.0	±	16.4
	5.02	--	51.0	±	4.2	417.0	±	134.9	468.0	±	134.3
	7.49	1	47.5	±	15.5	431.3	±	51.2	478.8	±	61.2
		2	35.0	±	24.8	1043.8	±	196.5	1078.8	±	200.1
	10.15	--	67.0	±	9.7	849.0	±	189.3	916.0	±	194.0
Blend 7	1.22	--	0.0	±	0.0	20.0	±	18.4	20.0	±	18.4
	2.45	--	0.0	±	0.0	36.0	±	15.6	36.0	±	15.6
	5.02	--	11.0	±	16.7	374.0	±	226.3	385.0	±	238.1
	7.49	1	141.3	±	58.4	321.3	±	106.8	462.5	±	72.9
		2	40.0	±	26.8	970.0	±	250.4	1010.0	±	255.6
	10.15	1	364.0	±	207.3	646.0	±	218.9	1010.0	±	259.4
		2	315.0	±	122.2	Unknown			No Steady State Film		
